# Accuracy of Monocular Two-Dimensional Pose Estimation Compared With a Reference Standard for Kinematic Multiview Analysis: Validation Study

**DOI:** 10.2196/19608

**Published:** 2020-12-21

**Authors:** Oskar Stamm, Anika Heimann-Steinert

**Affiliations:** 1 Geriatrics Research Group Charité – Universitätsmedizin Berlin, corporate member of Freie Universität Berlin, Humboldt-Universität zu Berlin, and Berlin Institute of Health Berlin Germany

**Keywords:** 2D human pose estimation, motion capturing, kinematics, clinical practice, mobility, smartphone app, analysis

## Abstract

**Background:**

Expensive optoelectronic systems, considered the gold standard, require a laboratory environment and the attachment of markers, and they are therefore rarely used in everyday clinical practice. Two-dimensional (2D) human pose estimations for clinical purposes allow kinematic analyses to be carried out via a camera-based smartphone app. Since clinical specialists highly depend on the validity of information, there is a need to evaluate the accuracy of 2D pose estimation apps.

**Objective:**

The aim of the study was to investigate the accuracy of the 2D pose estimation of a mobility analysis app (Lindera-v2), using the PanopticStudio Toolbox data set as a reference standard. The study aimed to assess the differences in joint angles obtained by 2D video information generated with the Lindera-v2 algorithm and the reference standard. The results can provide an important assessment of the adequacy of the app for clinical use.

**Methods:**

To evaluate the accuracy of the Lindera-v2 algorithm, 10 video sequences were analyzed. Accuracy was evaluated by assessing a total of 30,000 data pairs for each joint (10 joints in total), comparing the angle data obtained from the Lindera-v2 algorithm with those of the reference standard. The mean differences of the angles were calculated for each joint, and a comparison was made between the estimated values and the reference standard values. Furthermore, the mean absolute error (MAE), root mean square error, and symmetric mean absolute percentage error of the 2D angles were calculated. Agreement between the 2 measurement methods was calculated using the intraclass correlation coefficient (ICC[A,2]). A cross-correlation was calculated for the time series to verify whether there was a temporal shift in the data.

**Results:**

The mean difference of the Lindera-v2 data in the right hip was the closest to the reference standard, with a mean value difference of –0.05° (SD 6.06°). The greatest difference in comparison with the baseline was found in the neck, with a measurement of –3.07° (SD 6.43°). The MAE of the angle measurement closest to the baseline was observed in the pelvis (1.40°, SD 1.48°). In contrast, the largest MAE was observed in the right shoulder (6.48°, SD 8.43°). The medians of all acquired joints ranged in difference from 0.19° to 3.17° compared with the reference standard. The ICC values ranged from 0.951 (95% CI 0.914-0.969) in the neck to 0.997 (95% CI 0.997-0.997) in the left elbow joint. The cross-correlation showed that the Lindera-v2 algorithm had no temporal lag.

**Conclusions:**

The results of the study indicate that a 2D pose estimation by means of a smartphone app can have excellent agreement compared with a validated reference standard. An assessment of kinematic variables can be performed with the analyzed algorithm, showing only minimal deviations compared with data from a massive multiview system.

## Introduction

Traditional movement assessments, although carried out by experienced physicians, physiotherapists, and occupational therapists, can contain inaccuracies due to subjectivity, despite the clinicians’ expertise. In contrast, quantitative motion measurements by motion capture systems are a valuable tool in scientific and clinical motion analysis and offer a highly accurate and reliable way of capturing kinematic data. Quantitative analyses can be used, for example, to monitor the progress of therapies and objectively evaluate the effectiveness of specific interventions. Motion capture systems are used in sports, biomechanics, and rehabilitation, and they focus on gait analysis, injury prevention, and performance improvement [1]. However, these systems are rarely used in everyday clinical practice.

There are many optoelectronic motion capture systems based on markers (eg, Vicon [Vicon Motion Systems], Motion Analysis [Motion Analysis Corp], Optitrack [NaturalPoint Inc], and Qualisys [Qualisys AB]). These systems are often regarded in the literature as the gold standard for motion capture [2]. Today, low- to high-speed experiments show that positioning errors can even be assumed in the case of measurements less than 2 mm [3]. Nevertheless, optoelectronic systems require a restricted area, such as a laboratory environment, and the attachment of markers [4], which can also be a potential source of measurement error in these systems due to skin movement artifacts [5]. Furthermore, these systems are proving to be very costly. The aforementioned factors are detrimental to the systems’ practicability and everyday use in a clinical context.

Inertial sensor measurement systems can be used as a low-cost alternative. However, an inertial sensor measurement system cannot determine global position when used as a stand-alone system (by itself), although as a fusion system, in combination with a rigid body model such as the Perception Neuron (Noitom Ltd), a position in space can still be identified [2]. Nevertheless, a major disadvantage remains in clinical practice, as users must attach numerous sensors to a patient’s body. Since clinical processes are usually efficiency driven and the application of several sensors is too time-consuming in most cases due to time constraints during treatment, this also contributes to the low frequency of use. Recently, studies have examined markerless and body sensor–less image-processing systems. Depth-sensing camera systems, such as the Kinect (Microsoft Corp), Intel RealSense (Intel Corp), or Zed (Stereolabs Inc) sensors, have proven to be a cost-effective solution with acceptable accuracy for some use cases [6-11]. Another low-cost motion capture method in image processing is pose estimation, which involves transformation of two-dimensional (2D) images into three-dimensional (3D) objects, for example, by using deep convolutional neural networks of monocular images [12-15], such as images on a mobile device [16]. Research into depth estimation from a monocular image is still in its infancy in the field of computer vision and is proving to be difficult in some cases, as slight inaccuracies in estimation can lead to very different depth estimates [17,18]. Major limitations include false pose estimates due to the target person moving outside the image boundaries [19] or the pose being disturbed by objects such as shadows [15].

A 2D skeleton detector enables calculation of specific joint angles for assessment and feedback in sport and rehabilitation settings. The use of 2D human pose estimations for clinical purposes, such as the Lindera-v2 app or the motion-tracking coach on the Kaia health app [20], allows mobility analyses to be carried out using, for example, a mobile device. Pose and movement analyses via a smartphone save time for medical staff, who can use the time gained as treatment time or for patient consultations. Furthermore, such a measurement of mobility represents a more objective method of measurement compared with traditional assessments, which are based on a subjective assessment. Since in clinical practice, specialist staff highly depend on the validity of information, there is a need to validate the methodology of the Lindera-v2 measuring method. In order to achieve a performance level comparable with the gold standard motion capture systems, this study aimed to evaluate the accuracy of the Lindera-v2 2D pose estimation algorithm, using the PanopticStudio Toolbox (Carnegie Mellon University) [21,22] as a reference standard.

## Methods

### Data Collection

For the accuracy evaluation, 10 video sequences were generated from Panoptic Studio 3D PointCloud (data set 171204_pose1-6) [21,22]. Data shared for research purposes from Carnegie Mellon University were used as the reference standard. The data set included video sequences from 480 video graphics array (VGA) cameras, 31 high-definition (HD) cameras, and 10 Kinect cameras, as well as arrays of 2D poses of key points of body part locations, showing the range of motion of the joints. The videos were split so that only one actor appeared in each video. Subsequently, 10 videos were selected that matched the requirement for all key joint points to be visible during the movements in each frame. Within the video clips, no changes or cuts were made. The total duration of the videos selected for analysis was 24 minutes 9 seconds, with an average duration of 2 minutes 25 seconds per video. The movements were categorized by the PanopticStudio Toolbox as a range of motion.

### Lindera Pose Estimation

**Figure 1:**
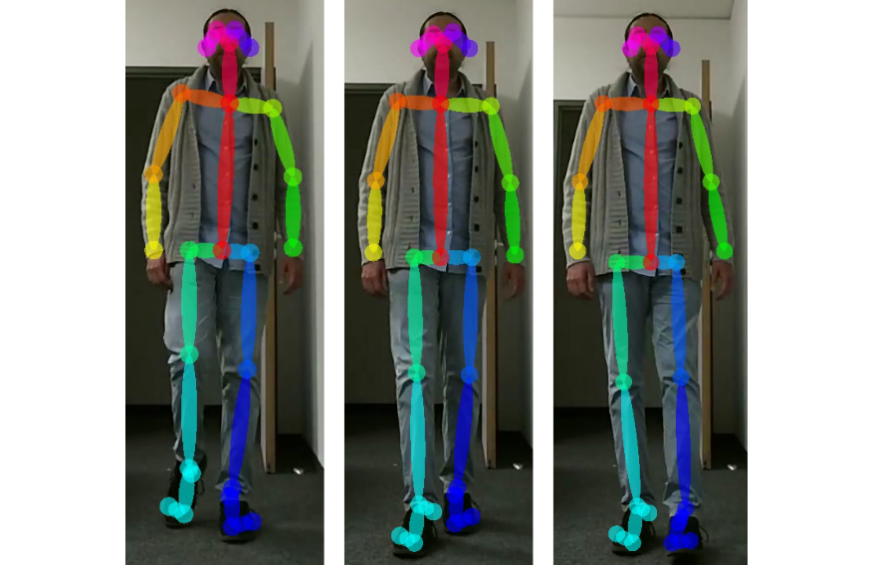
Two-dimensional pose estimation by skeleton fitting, based on 25 body joint coordinates.

The Lindera-v2 algorithm is a combination of a 2D and 3D skeleton-based pose estimation. For this study, we needed the output of the 2D skeleton estimator to calculate 2D joint angles [23,24]. The 2D skeleton detector module of the Lindera-v2 algorithm is based on the tf-pose-estimation repository [25]. This repository is a TensorFlow implementation of various deep learning models [13] that represent human pose estimation models based on convolutional pose machines [26]. The original repository also provides some model variants that run on mobile devices. The short version of the repository used with the Lindera-v2 algorithm has also implemented the additional Openpose Body25 model, which provides 25 body joint coordinates for each input image (*x* in the direction of the image width and *y* in the direction of the image height) instead of 18 body joint coordinates, as was the case in the original repository model variants (Figure 1). The 2D module of the Lindera-v2 algorithm produced a coordinate-time list for each joint and each frame. These time series data were then used to geometrically calculate the corresponding 2D joint angles for each frame of the processed input video. To smooth the angle-time series data, we used digital filtering with a size 11 Bartlett window.

### Reference Standard

The Panoptic Studio data set from Carnegie Mellon University [21,22] is a data set shared for research purposes. Unlike the Lindera app, the PanopticStudio Toolbox is not a monocular 2D estimator but rather a massive multiview system consisting of the following: (1) 480 VGA cameras with a resolution of 640 × 480 pixels and 25 frames per second (fps); (2) 31 HD cameras with a resolution of 1920 × 1080 pixels and 30 fps; (3) 10 Kinect Ⅱ sensors of 1920 × 1080 pixels (RGB), 512 × 424 pixels (depth), and 30 fps; and (4) 5 digital light processing projectors.

The 2D skeleton of the 2D Panoptic Studio pose detector has 15 anatomical landmarks. The 2D detector uses appearance information in the interpretation and includes connectivity information.

### Statistical Analysis

The value tables for the respective joint angles were clustered, and missing values were imputed using a simple moving average. The mean difference (bias) between the Lindera-v2 algorithm estimates and the reference standard values was calculated for each joint. Furthermore, the mean absolute error, the root mean squared error, and the symmetric mean absolute percent error of the 2D angles were used. The intraclass correlation coefficient (ICC[A,2]) was calculated for the data using the 2-way mixed-effects model as a measure of agreement between the 2 measurement methods. An ICC in the range of 0 indicates random evaluation behavior, and a value of 1 is regarded as an ideal reliable feature evaluation by the evaluators. We used the definition in which values greater than 0.7 are generally regarded as indicators of good agreement [27]. Values up to approximately 0.3 are regarded as a low correlation, and those of approximately 0.5 or more are regarded as a medium correlation. A further classification according to Fleiss [28] was used to assesses the ICC classification, with 0.00 to 0.40 indicating poor agreement, 0.40 to 0.75 indicating fair to good agreement, and 0.75 to 1.00 indicating excellent agreement.

A cross-correlation was calculated for the time series to verify whether there was a temporal shift in the data. To verify the stationarity of the data, which is a prerequisite for cross-correlation testing, we used the augmented Dickey-Fuller test. The data were first evaluated in IBM SPSS Statistics (version 25.0; IBM Corp) and then in the programming language R in RStudio (version 3.5.1; RStudio Inc).

## Results

In order to evaluate the accuracy of the movement signals recorded, we analyzed a total of 30,000 data pairs for each joint, comparing the joint angles obtained using the Lindera-v2 algorithm with those of the PanopticStudio Toolbox data set (the reference standard). Table 1 displays the 10 joints analyzed; the key points used for calculating the joint angles; the average difference (bias) between the estimated values of the Lindera-v2 algorithm and the reference standard; and the mean absolute error (MAE), mean absolute deviation, root mean square error (RMSE), symmetric mean absolute percentage error (sMAPE), and ICC. The angles used were determined trigonometrically for both measuring methods.

**Table 1:** Mean angle difference and ICC of Lindera-v2 and the Panoptic Studio data set for the joints analyzed.

Joint	2D^a^ key points used	Difference in 2D angles (°), mean (SD); 95% CI	MAE^b^ of 2D angles (°)	MAD^c^ (°)	RMSE^d^ of 2D angles (°)	sMAPE^e^ (%)	ICC^f^ (95% CI)	SE of mean difference
Right shoulder	Right hip, shoulder, and elbow	2.71 (10.28); 2.59 to 2.83	6.48	4.10	10.63	23.33	0.978 (0.973 to 0.981)	0.06
Left shoulder	Left hip, shoulder, and elbow	–0.07 (12.11); –0.21 to 0.07	3.98	3.20	12.12	10.71	0.951 (0.950 to 0.952)	0.07
Right elbow	Right shoulder, elbow, and wrist	–1.01 (12.12); –1.15 to –0.87	6.18	4.30	12.16	6.64	0.983 (0.983 to 0.984)	0.07
Left elbow	Left shoulder, elbow, and wrist	0.24 (6.20); 0.17 to 0.31	3.15	2.84	6.21	9.17	0.997 (0.997 to 0.997)	0.04
Right hip	Right shoulder, hip, and knee	–0.05 (6.06); –0.12 to 0.02	4.45	4.68	6.06	3.01	0.983 (0.983 to 0.983)	0.04
Left hip	Left shoulder, hip, and knee	–0.61 (3.85); –0.66 to –0.57	2.29	2.29	3.90	1.74	0.992 (0.992 to 0.993)	0.02
Right knee	Right hip, knee, and ankle	–1.37 (2.97); –1.40 to –1.34	2.58	2.93	3.27	1.56	0.985 (0.974 to 0.990)	0.02
Left knee	Left hip, knee, and ankle	0.84 (4.31); 0.79 to 0.89	2.28	2.45	4.44	1.39	0.971 (0.968 to 0.974)	0.03
Neck	Pelvis, neck, and head	–3.07 (6.43); –3.14 to –2.99	4.47	3.63	7.13	3.20	0.951 (0.914 to 0.969)	0.04
Pelvis	Left knee, pelvis, and right knee	0.15 (2.03); 0.14 to 0.18	1.40	1.64	2.04	5.42	0.996 (0.996 to 0.996)	0.01

^a^2D: two-dimensional.

^b^MAE: mean absolute error.

^c^MAD: mean absolute deviation.

^d^RMSE: root mean square error.

^e^sMAPE: symmetric mean absolute percentage error.

^f^ICC: intraclass correlation coefficient ICC(A,2).

The data collected indicated both a negative and a positive bias. The mean difference of the joint angles that was nearest to the baseline was identified in the right hip (–0.05°, SD 6.06°). The joint with the highest mean difference (ie, with the greatest difference from 0) was the neck (–3.07°, SD 6.43°). The mean joint angle accuracy was used to show the average magnitude of the errors. The mean absolute error of the angle measurement closest to the baseline was observed in the pelvis (1.40°, SD 1.48°). In contrast, the highest mean absolute error was observed in the right shoulder (6.48°, SD 8.43°). The standard deviation was also lowest in the pelvis (SD 3.36°), and the highest standard deviation was found to be in the left shoulder (SD 11.45°). The root mean square error was also applied, although this tends to give weight to large errors. The RMSE indicated low accuracy in the right elbow (12.16°) and high accuracy in the pelvis (2.04°). Since the mean absolute percentage error cannot be used when values are 0 (as this would result in division by 0), we used the sMAPE, which was lowest in the left knee (1.39%) and highest in the right shoulder (23.33%).

The intraclass correlation coefficient for the joint angles is also shown in Table 1 and represents agreement between the 2 measurement methods (Lindera-v2 vs the PanopticStudio Toolbox). In accordance with the McGraw and Wong convention [29], the intraclass correlation coefficient ICC(A,2) was used (ie, a 2-way mixed type with average measures and absolute agreement). The highest ICC value was found in the left elbow joint (average measure of 0.997, 95% CI 0.997-0.997). In contrast, the lowest ICC values were in the neck (average measure of 0.951, 95% CI 0.914-0.969).

**Figure 2:**
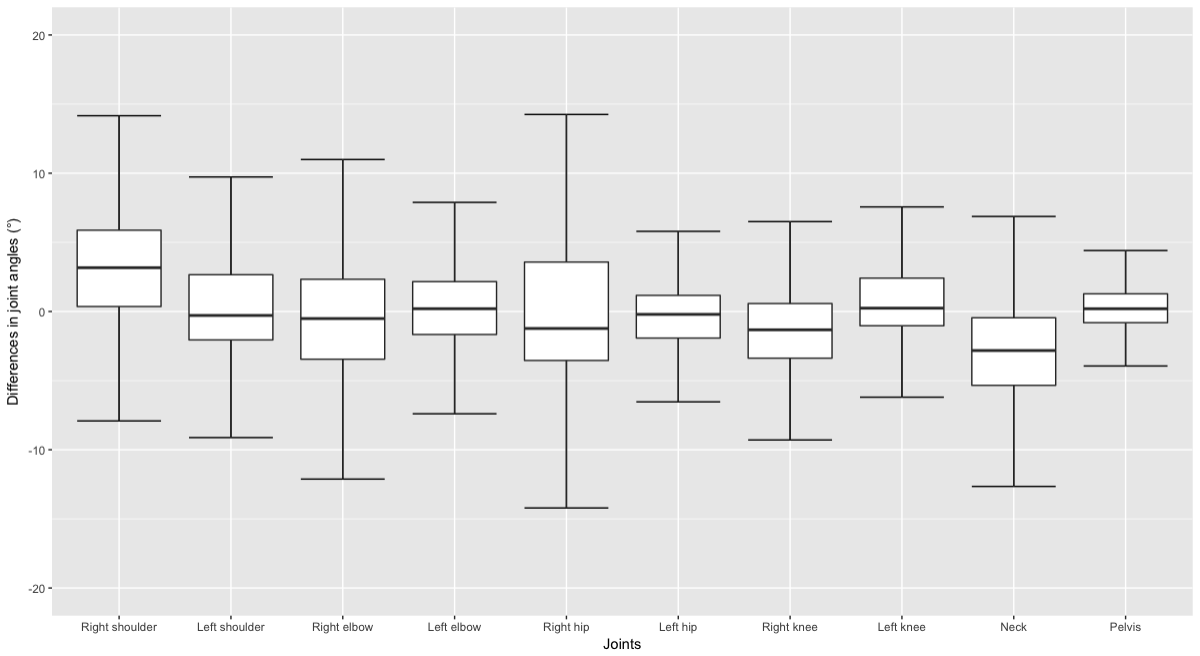
Box plot showing differences in the Lindera-v2 and reference standard values, measured across all joints tested.

**Figure 3:**
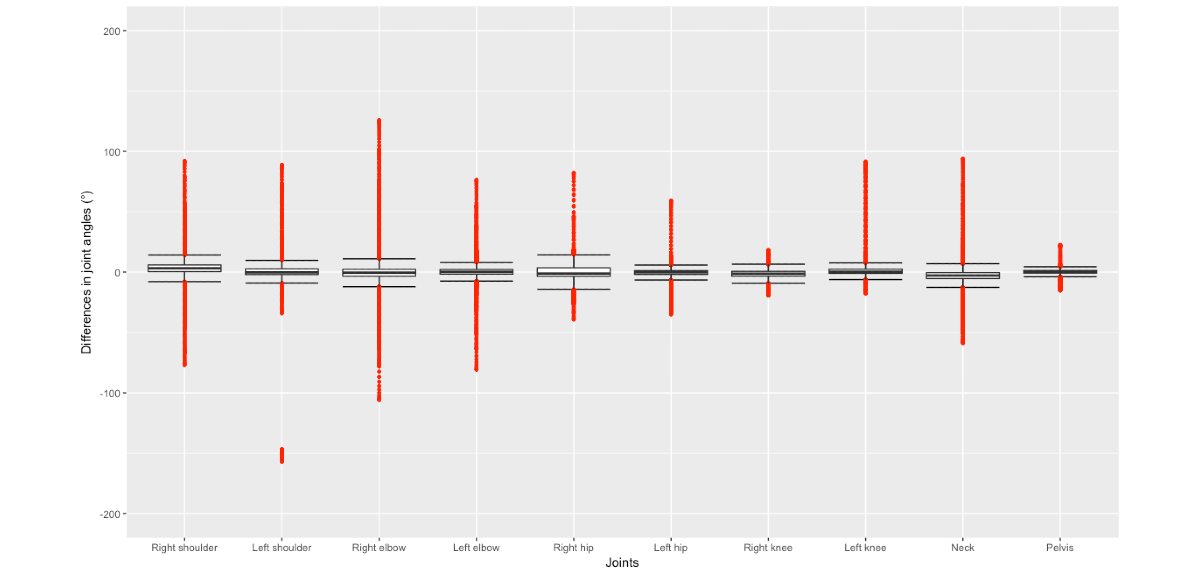
Box plot with outliers showing differences in the Lindera-v2 and reference standard values, measured across all joints tested.

Interpretation of the measurement values based on mean values can lead to biased findings (eg, in the case of extreme outliers). Since the median is less affected by outliers, we used box plots for the differences in joint angle values measured with the Lindera-v2 and the reference standard. Figure 2 shows a box-and-whisker plot without outliers to facilitate closer examination of the boxes. The ends of the whiskers represent 1.5 × IQR. The median with the greatest difference in comparison with the 0 value was detected in the right shoulder (3.17°), and the joint angle median nearest to the baseline was the pelvis joint (0.19°). Figure 3 shows the box-and-whisker plot with outliers. The third quartile of the right shoulder was the farthest from the baseline of all the joints, with a value of 5.87°. The lowest first quartile was in the neck, measuring –5.34°. The smallest IQR, ranging from –0.81° to 1.27°, was in the pelvis. The most extreme outliers in this plot were found in the right elbow, where the minimum was –106.00° and the maximum was 125.71°. However, the outlier with the greatest difference in comparison to 0 was in the left shoulder, with a difference of 157.10°. 

**Figure 4:**
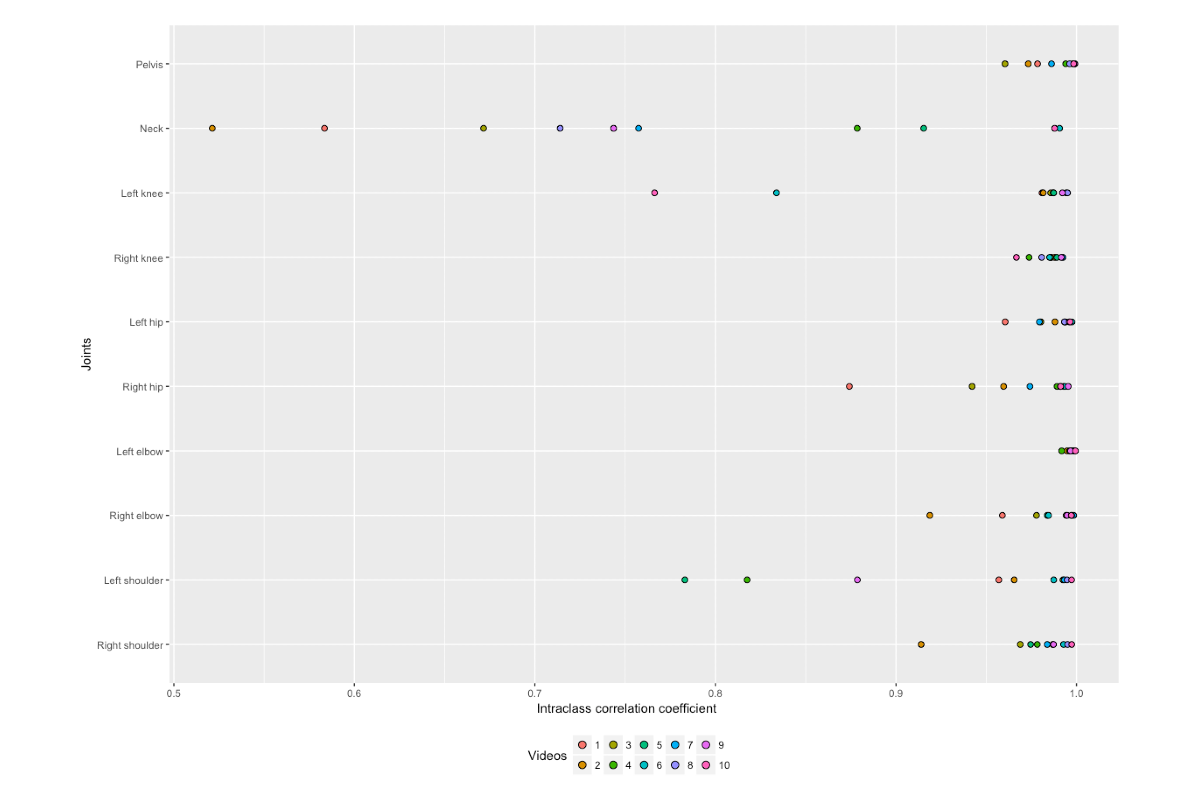
Dot plot of the intraclass correlation coefficient comparing Lindera-v2 and reference standard for the 10 single videos used for accuracy measurement in each joint.

To examine the ICC more closely and analyze the potential influence of single videos on the ICC values of the joints, our next step entailed calculating an ICC for each video. Figure 4 shows a dot plot of the ICC for the 10 videos used for the accuracy measurement in each joint. For the neck joint, 5 videos had an ICC below 0.75. However, there were no videos in which all joints had remarkably low ICC values. With the exception of the neck, all joints in all videos had an ICC value above 0.75.

**Figure 5:**
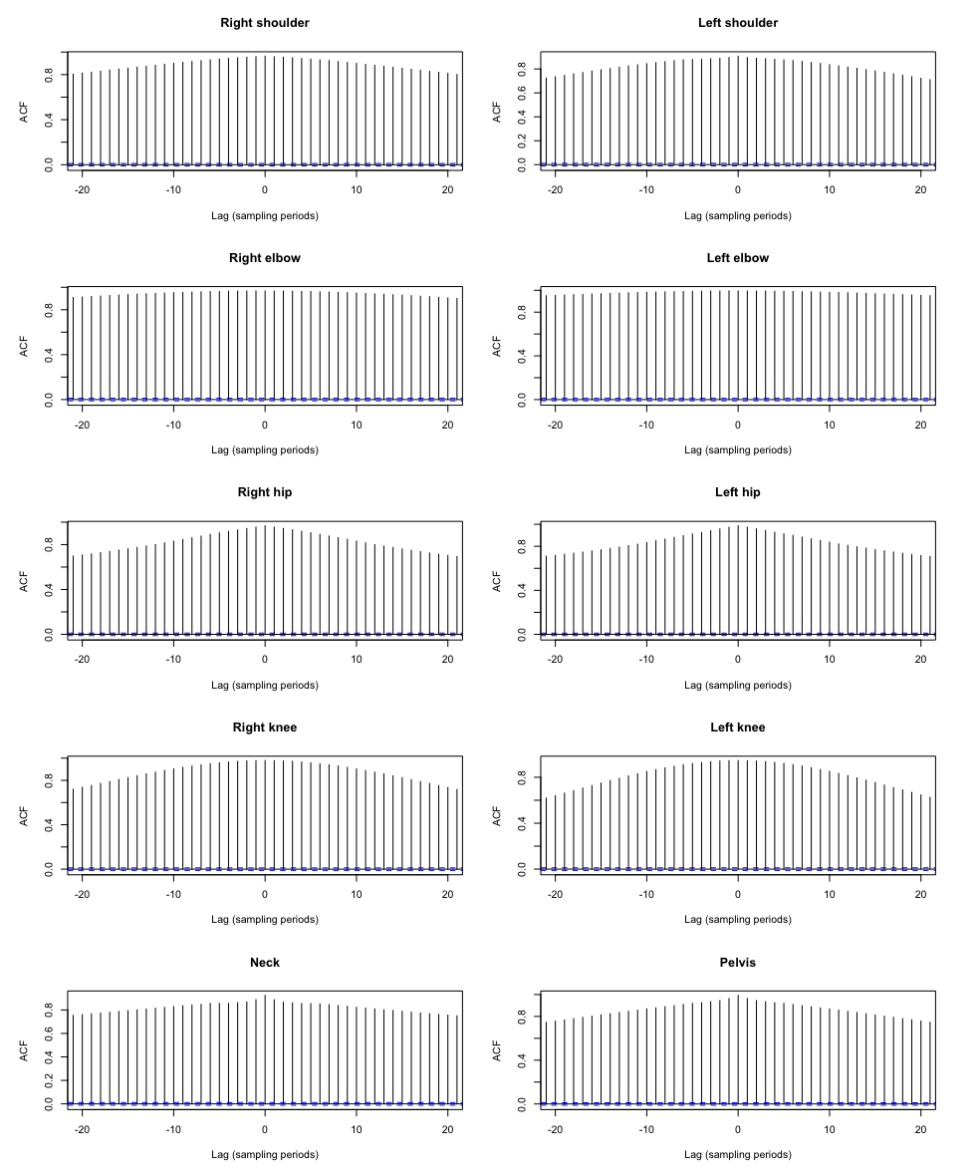
Cross-correlation graph of Lindera-v2 and Panoptic Studio data set values. One lag represents 1 sample (frame). ACF: autocorrelation function.

The cross-correlation function was applied to the selected time series in order to examine the temporal lag. The results in Table 2 show that no time delays could be detected in the values measured. These would have been visible at an increased correlation at a time outside lag 0. However, all graphs (Figure 5) showed the highest correlation at lag 0. The dotted blue lines represent the confidence interval of the estimated correlation values. If a value was outside the range of the interval, the correlation was considered significant.

**Table 2:** Maximum cross-correlations of Lindera-v2 and reference standard values.

Joint	Lag value with maximum correlation	Maximum correlation coefficient
Right shoulder	0	0.96
Left shoulder	0	0.91
Right elbow	0	0.97
Left elbow	0	0.99
Right hip	0	0.97
Left hip	0	0.99
Right knee	0	0.98
Left knee	0	0.95
Neck	0	0.92
Pelvis	0	0.99

## Discussion

### Principal Results

The goal of this study was to validate the accuracy of the 2D pose estimation of joint angles obtained from the Lindera-v2 algorithm, using the PanopticStudio Toolbox, which served as the reference standard. Therefore, we analyzed 30,000 data pairs for each joint angle during diverse total body motion activity. First, the mean difference and error measures were compared for each joint. Second, the ICC was compared for each joint. In order to verify agreement between the 2 measurement methods (the Lindera-v2 and the PanopticStudio Toolbox data set), we analyzed the ICC values for each of the 10 videos. Finally, we examined the potential temporal lag through cross-correlation. The results of the study indicate that the 2D pose estimation method used had excellent agreement with the reference standard. Furthermore, the Lindera-v2 algorithm had no temporal lag.

The mean angle generated for the right hip by the Lindera-v2 algorithm was the closest to the reference standard. Even the value with the greatest difference from 0 (found in the neck) was acceptable. However, these values should be treated with caution because mean values can lead to biased results. Therefore, we displayed the median values in box plots. The medians of all joints compared with the reference standard ranged from a difference of 0.19° (pelvis joint) to 3.17° (right shoulder). In all joints, the IQR was within 6° and –6°, which means that 50% of the values were within this range. These acquired values provide a promising starting point upon which to base mobility assessments and 3D pose estimation. A further reason why box plots were used was to identify outliers because the RMSE used gives greater weight to large errors. Figure 3 shows that in the box-and-whisker plots, the data recording the difference between the Lindera-v2 algorithm and the reference standard had few very high outliers. In the right elbow, for example, the outliers were particularly high compared with the other joints. Since the RMSE squares the errors before averaging, the RMSE in this joint was the highest. Since large outliers can be quickly identified as such by an experienced user, the weighting of large outliers by the RMSE does not seem appropriate. Hence, the MAE might be the more appropriate measure. The sMAPE is more resistant to outliers due to defined error limits because it gives less weight to outliers than other measures that have no error limits; it was applied additionally for this reason [30]. An advantage over the mean absolute percentage error is that the sMAPE cannot be extremely large or infinite [30]. The sMAPE in our evaluation was particularly high in the right shoulder (23.33%). Possible joint “losses” could be an explanation due to bad visibility of the joints in the videos, perhaps due to the clothing of the participant or the lighting used.

The ICC agreement between the 2 measurement methods can be interpreted as excellent (according to the classification presented by Fleiss [28]). All joints had an ICC value of at least 0.951. Furthermore, the 95% confidence interval of the ICC for all joints can be classified as excellent. Our analysis of the individual videos showed why the neck had the lowest overall agreement in comparison with the other joints. In several videos, the agreement could be interpreted as fair to good. We assume that this relates to the approximations of neck positions, since these were calculated from the key point of the nose in the Lindera algorithm. By applying cross-correlation, angles estimated through the Lindera-v2 algorithm showed no temporal lag.

Early research and reviews published in 2016 reported that the Kinect skeleton-tracking algorithm indicated poor validity and large errors with respect to most kinematic variables [7]. Clark et al [10] recommended that the Kinect system be carefully chosen for specific use cases (eg, trunk angles can be highly accurate). An extensive recent review by Poitras et al [31] on the validity and reliability of wearable sensors for joint angle estimation revealed mixed results. The results presented in this study are therefore very promising, not only because of the acceptable accuracy of the angles but also because the usability of smartphone apps (compared with the Kinect system or wearable sensors) offers major advantages. Schurr et al [32] showed a moderate to strong relationship between a 2D video camera and 3D motion capture analyses. From this point of view, 2D pose estimations are applicable in clinical practice. Even though 2D cameras offer clinicians a valuable kinematic measurement tool, the use of a smartphone would be far less complicated and would make the technology available to a wide user group.

Valid and reliable 2D joint angles are an important first step on the way to valid and reliable 3D joint angles. Therefore, in the next step, the 2D data from the evaluation will be transformed into 3D pose estimation angles using deep convolutional neural networks. A validation of the 3D joint angle accuracy of the resulting data will show whether the requirements for clinical practice are met.

### Limitations

Although this study showed excellent agreement to a reference standard, a validity study using a state-of-the-art marker-based motion capture system as a ground truth is necessary for a thorough validation. The comparison to the reference standard is an important step toward accuracy assurance but does not replace a proof of validity.

To determine a systematic error in the algorithm by an offset, a static setup would be needed. From this, a Euclidean distance could be calculated to identify a precise source of error. The mean joint position error is the most frequently used method for verification of the accuracy of a pose estimation. However, since determination of the coordinates in millimeters in space was not possible in these data sets, accuracy verification was carried out for the joint angles. Verification of the precision showing the repeatability of the data was not planned in this project, since measurement using the Lindera-v2 was carried out once and the movements were not repeated in a standardized manner. However, the precision of the time stamps within the measurement of the evaluated movements can be seen from the standard errors of the mean difference. A validation of the precision will be the subject of further studies.

### Perspectives

In geriatrics, orthopedics, and neurology in particular, accurate and validated mobility analyses such as the Lindera-v2 could help medical professionals confirm diagnoses and track the success of treatments. Mobility assessments have very high relevance for a multitude of clinical uses (eg, older adults and patients with more severe diseases who have a higher risk of falling) [33,34]; in this case, fall risk assessments could be of high value. Assessment of kinematic variables, such as specific joint angles, can be accessed via 2D skeleton data if viewed from specific angles, and such data can also be used for rehabilitative purposes in physical therapy or sports science. Furthermore, the 2D values analyzed in this study constitute an encouraging basis for 3D pose estimation, which will be the next step in accuracy validation.

### Conclusions

The results of the study indicate that 2D pose estimation by means of a camera-based smartphone app can have excellent agreement with a validated reference standard. Furthermore, the Lindera-v2 algorithm was found to have no temporal lag. An assessment of kinematic variables, such as specific joint angles, can be performed with the algorithm, and these data showed only minimal deviations compared with data from a massive multiview system. In future studies, it will be important to test the app in a clinical context with participants with physical limitations.

## Abbreviations

2D: two-dimensional
3D: three-dimensional
fps: frames per second
HD: high definition
ICC: intraclass correlation coefficient
MAE: mean absolute error
RMSE: root mean square error
sMAPE: symmetric mean absolute percentage error
VGA: video graphics array
